# Protective Effect of Obeticholic Acid on Sepsis-Induced Liver Dysfunction via Regulating Bile Acid Homeostasis

**DOI:** 10.3390/ph18050763

**Published:** 2025-05-21

**Authors:** Jiahui Wang, Li Ma, Yuan An, Yan Ge, Dan Xu, Enqiang Mao

**Affiliations:** 1 Department of Emergency, Ruijin Hospital, Shanghai Jiao Tong University School of Medicine, Shanghai 200025, China; wjh12997@rjh.com.cn (J.W.); ml11948@rjh.com.cn (L.M.);; 2 Department of Orthopaedics, Shanghai Key Laboratory for Bone and Joint Diseases, Shanghai Institute of Traumatology and Orthopaedics, Ruijin Hospital, Shanghai Jiao Tong University School of Medicine, Shanghai 200025, China

**Keywords:** sepsis, liver injury, bile acids, FXR, inflammatory response

## Abstract

**Background/Objectives:** Abnormal bile acid (BA) pool may play an important role in inducing liver damage in sepsis. Farnesoid X receptor (FXR) is a main negative feedback regulator of BA metabolism. This study aims to explore the protective effect and mechanism of the FXR agonist obeticholic acid (OCA) on liver dysfunction when sepsis occurs. **Methods:** A rat model of sepsis was induced by cecal ligation and puncture (CLP) for 24 h. Systematic inflammation, tissue injury, hepatic FXR, and BA transporter expression were investigated in the CLP rats and sham-operated control rats with and without OCA pre-treatment (10 mg/kg, gavage) at 2 h before operation. Liquid chromatography–tandem mass spectrometry (LC-MS/MS) assay was performed to access BA composition in the rats’ serum and livers. The injury and inflammatory effects of the elevated unconjugated BAs found in the CLP rats was further verified in a hepatic cell line BRL-3A in vitro. **Results:** Hepatic FXR was repressed in CLP rats, whereas OCA upregulated liver FXR and hepatic BA transporter expression, reduced total serum BA concentration, ameliorated the elevation of serum levels of IL-1β and IL-6, and improved liver and ileal tissue injuries. OCA administration reduced the elevated unconjugated BAs in both serum and liver, and effectively inhibited increases in cholic acid (CA), deoxycholic acid (DCA), and 7-ketoDCA concentrations in CLP rat livers. These BA fractions promoted the release of aspartate aminotransferase (AST) from BRL-3A cells and increased IL-6, CXCL2, and monocyte chemoattractant protein-1 (MCP-1) expression in the cells, along with enhanced transcription factor nuclear factor-κB activation. **Conclusions:** Liver inflammation and dysfunction during sepsis is attributable to significant changes in bile acid composition in the blood and liver. FXR activation reduces systemic inflammation and liver dysfunction by regulating bile acid homeostasis, especially inflammatory unconjugated bile acid components.

## 1. Introduction

Sepsis is a life-threatening organ dysfunction caused by a dysregulated host response to infection [1]. Among all the organ failures that might occur during sepsis, liver dysfunction is the most prognosis-related and a strong risk factor of mortality [2]. Hypoxic hepatitis and sepsis-induced cholestasis are two main clinical features when liver dysfunction occurs in sepsis [3]. Nevertheless, the pathophysiology of sepsis-induced liver dysfunction is still not well understood. Organ crosstalk, especially gut–liver crosstalk between the gut microbiota and bile acid (BA) metabolism and its signaling pathway, might play an important role [4].

BAs are a group of structurally diverse molecules biosynthesized in the liver and transformed by the gut microbiota. They are essential for lipid digestion, energy metabolism, and protection against bacteria overgrowth [5,6], and are involved in various liver, biliary, and intestinal diseases. Primary BAs, i.e., cholic acid (CA) and chenodeoxycholic acid (CDCA), are synthesized in the liver, conjugated with glycine or taurine in hepatocytes, secreted into bile via the bile salt export pump (BSEP), and expelled in the intestine [7]. In the terminal ileum, primary conjugated BAs are converted into secondary BAs by the gut microbiota, which include deoxycholic acid (DCA), lithocholic acid (LCA), and ursodeoxycholic acid (UDCA). The majority of BAs are absorbed into enterocytes, transported through the basolateral membrane, enter the portal vein, and are finally reabsorbed into hepatocytes via Na^+^-taurocholate transporting polypeptide (NTCP) and organic anion-transporting polypeptides (OATPs) [8]. Approximately 95% of BAs are reabsorbed into the liver through the gut–liver axis [9]. Excessive levels of BAs may activate inflammatory pathways and kill hepatocytes via multiple pathways, including apoptosis, necrosis, and pyroptosis [10,11]. BA toxicity depends greatly on their hydrophobicity and conjugation status, which determines the ability of BAs to penetrate cell membranes [12,13]. Hydrophobic BAs activate inflammation response by stimulating pro-inflammatory mediator production, such as cytokines, chemokines, and adhesion molecules [14,15]. During sepsis, cholestasis and circulating bile acid accumulation are common and early symptoms [2,16,17,18]. Individual and total BAs are elevated by various degrees in different shock conditions. BAs represented an early predictor of short-term survival in a mixed cohort of ICU patients and may serve as a marker for early risk stratification in critically ill patients [19]. Recent clinical metabolomic studies further demonstrated strong links between hydrophobic BAs such as glycochenodeoxycholate (GCDCA) and taurocholic acid (TCA) and survivorship in septic patients [20,21], while less is known about how the BA pool changes and whether bile acid alteration may provoke liver injury in sepsis. Regulating bile acid metabolism might be a potential intervention target for liver injury in sepsis.

Farnesoid X receptor (FXR) is a crucial bile acid-regulating nuclear receptor, which participates in almost every aspect of bile acid physiology [22]. Upon ligand binding, FXR regulates gene expression by binding specific DNA motifs named FXR response elements (FXREs) that are localized in the promoter and intron regions of targeted genes [23,24]. When cholestasis occurs, FXR upregulates the expression of small heterodimer partner (SHP), leading to transcriptional repression of bile acid synthesis and hepatocyte BA intake, while enhancing the expression of BA export [23,25]. FXR activation also has anti-inflammatory effects in both chronic and acute liver diseases [26,27]. However, clinical research indicates that liver FXR is reduced in critically ill patients [2,28]. The role of FXR in sepsis-induced liver injury requires further delineation. Obeticholic acid (OCA), also referred to as 6-ethyl-chenodeoxycholic acid, is a potent semisynthetic CDCA-derived FXR agonist [29]. It has shown its potential treatment effect in several chronic liver diseases [26,27], and obtained FDA approval as a second-line treatment for primary biliary cholangitis (PBC) [30]. However, the effectiveness of OCA on FXR activation and BA regulation in sepsis-induced liver injury has been poorly studied.

The aim of this study was to investigate BA alteration and its impact on liver injury in sepsis, and the effect of OCA treatment on liver dysfunction and BA metabolism when sepsis occurs.

## 2. Results

### 2.1. FXR Agonist OCA Upregulates Hepatic FXR in CLP Rats

**Figure 1:**
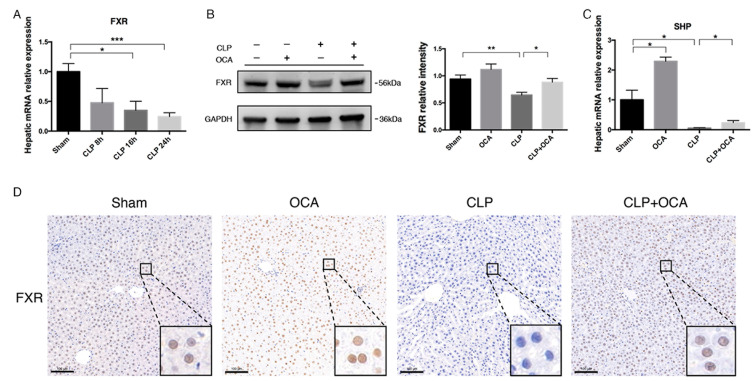
OCA stimulates the expression of FXR and SHP in rat liver. (**A**) Time-dependent relative mRNA expression levels of FXR at 8 h, 16 h, and 24 h after CLP treatment. Furthermore, rats were divided into 4 groups: the sham group, OCA group, CLP group, and CLP + OCA group. Liver tissues were collected 24 h after CLP treatment. (**B**) Representative WB analysis of FXR (**left**) and relative protein levels of FXR (**right**) in each group of rats. (**C**) The mRNA level of SHP. (**D**) Immunochemistry staining of hepatic FXR, scale bars = 100 μm. Abbreviations: FXR, Farnesoid X receptor; SHP, small heterodimer partner; Sham, sham operation group; OCA, sham operation pre-treated with 10 mg/kg obeticholic acid; CLP, cecal ligation and puncture; CLP + OCA, CLP rats pre-treated with 10 mg/kg OCA. * *p* < 0.05; **, *p* < 0.01; *** *p* < 0.001, *n* = 6 rats/group.

In a rat model of sepsis induced by cecal ligation and puncture (CLP), we first studied the mRNA expression of hepatic FXR and the impact of OCA administration prior to CLP operation on the alteration of hepatic FXR expression and activation. It was found that the mRNA level of FXR decreased in a time-dependent manner in septic rats induced by CLP operation (Figure 1A). By Western blot and immunochemistry staining, it was revealed that OCA pre-treatment significantly increased the protein levels of FXR in the liver at 24 h after CLP operation (Figure 1B,D). The mRNA level of hepatic small heterodimer partner (SHP), a main downstream nuclear receptor regulated by FXR, was significantly downregulated after CLP operation. However, the level was 4-fold higher at 24 h after CLP operation in the OCA pre-treated rats compared with the CLP rats (Figure 1C). These data indicated an impairment of hepatic BA metabolism in CLP rats and an ameliorating effect of OCA on it.

### 2.2. Hepatic FXR Activation Ameliorates Inflammation and Tissue Injury in CLP Rats

**Figure 2:**
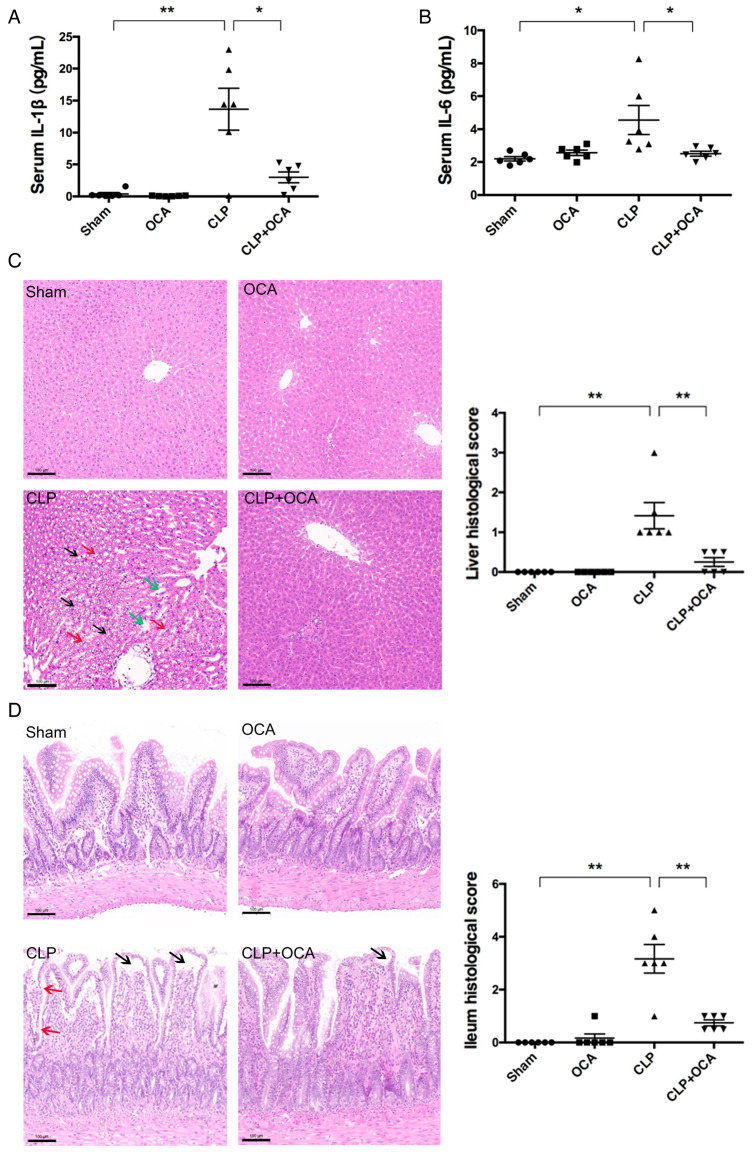
Hepatic FXR activation with OCA treatment reduces CLP-induced systematic inflammation and enterohepatic injury. Serum (**A**) IL-1β and (**B**) IL-6 levels were determined by ELISA in sham group, OCA group, CLP group, and CLP + OCA group 24 h after CLP. (**C**) Hepatic and (**D**) intestinal histopathology was shown by H&E staining and histological score was evaluated as described in methods, scale bars = 100 μm. (**C**) Cytoplasmic vacuolation (black arrows), focal to extensive nuclear pyknosis (red arrows), and sinusoid expansion (green arrows) were observed in liver sections of CLP group. (**D**) Intestine sections of CLP group showed extensive epithelial lifting (black arrows) and villi disintegration (red arrows). OCA did not change hepatic and intestinal histology in sham rats, but attenuated tissue damage induced by CLP administration. *, *p* < 0.05; **, *p* < 0.01, *n* = 6.

To evaluate the effect of hepatic FXR activation on systematic inflammation, serum IL-1β and IL-6 were detected in serum samples collected in rats of each group at 24 h after CLP. As shown in Figure 2A,B, rat serum levels of IL-1β and IL-6 were elevated at 24 h after CLP operation, indicating a successful model establishment. Hepatic FXR activation with OCA significantly decreased the elevated serum levels of IL-1β and IL-6 in CLP rats to near the levels of sham-operated rats. By histological analysis on H&E staining slides with scoring systems, it was shown that hepatic FXR activation with OCA reduced liver and intestinal injuries in CLP rats (Figure 2C,D).

### 2.3. Hepatic FXR Activation Modulate Liver Function and Bile Acid Metabolism in CLP Rats

**Figure 3:**
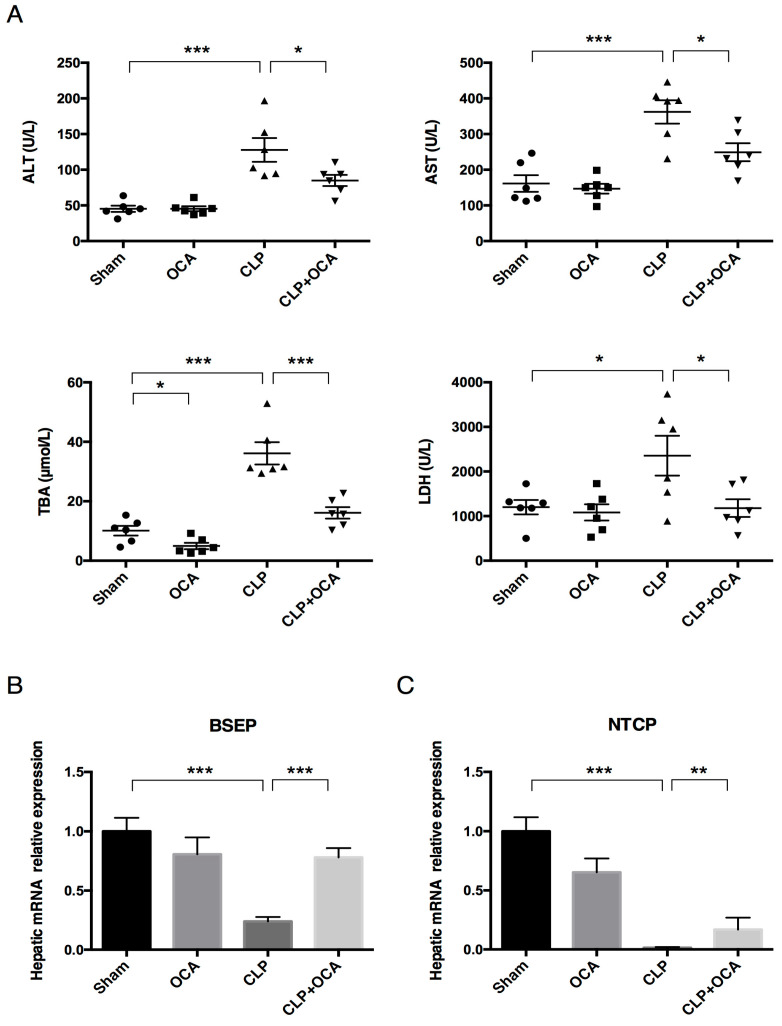
Hepatic FXR activation normalizes serum liver function indicators and improves the expressions of liver NTCP and BSEP in CLP rats. (**A**) Serum ALT, AST, TBA, and LDH levels and hepatic mRNA level of (**B**) BSEP and (**C**) NTCP in the sham group, OCA group, CLP group, and CLP + OCA group 24 h after CLP. Abbreviations: ALT, alanine aminotransferase; AST, aspartate aminotransferase; TBA, total bile acid; LDH, lactate dehydrogenase; BSEP, bile salt export pump; NTCP, Na^+^-taurocholate transporting polypeptide. *, *p* < 0.05; **, *p* < 0.01; ***, *p* < 0.001, *n* = 6.

To assess the effect of hepatic FXR activation on the liver dysfunction of CLP rats, rat serum ALT, AST, total bile acid (TBA), and lactate dehydrogenase (LDH) were measured at 24 h after CLP operation with or without pre-administration of OCA. CLP treatment significantly increased serum ALT, AST, TBA, and LDH levels, whereas FXR activation reduced the elevation of these serum biochemical indicators of liver function in CLP rats (Figure 3A). To verify how FXR activation decreases serum TBA levels, we assessed the mRNA level of BSEP and NTCP in rat livers. Liver NTCP and BSEP mRNA levels were significantly decreased in CLP rats whereas OCA pre-treatment reversed their downregulation by CLP operation (Figure 3B,C), indicating that FXR activation may improve BA excretion into the biliary duct and promoted hepatocyte BA uptake in the livers of CLP rats.

### 2.4. Hepatic FXR Activation Maintains BA Homeostasis in CLP Rats

**Figure 4:**
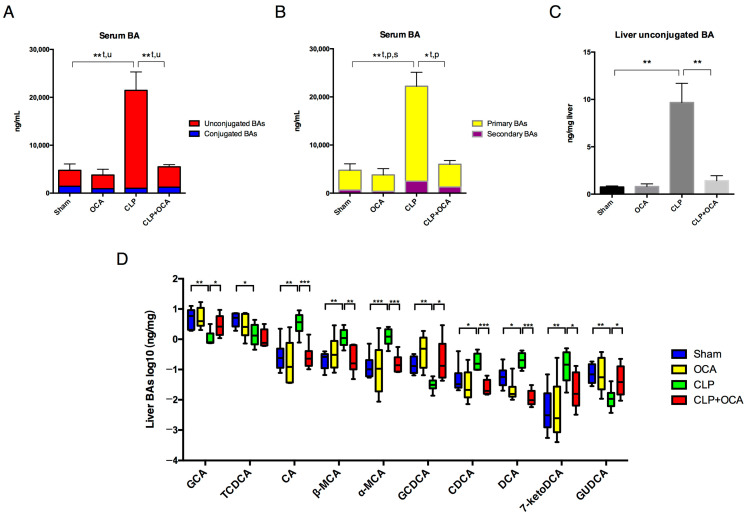
Hepatic FXR activation ameliorated altered serum and liver BA profiles in CLP rats. Serum bile acid levels categorized by (**A**) conjugation and (**B**) primary/secondary BAs in sham group, OCA group, CLP group, and CLP + OCA group 24 h after CLP. (**C**) Liver unconjugated BA levels in four groups. (**D**) Differences in liver BAs in four groups by Kruskal–Wallis test. Abbreviations: t, total; u, unconjugated; p, primary; s, secondary. *, *p* < 0.05; **, *p* < 0.01; ***, *p* < 0.001.

To further investigate how FXR activation improves bile acid metabolism in CLP induced sepsis, bile acid composition in livers and serums was analyzed using mass spectrometry. In accord with the serum biochemical analysis, total serum bile acid concentration was elevated in CLP rats and the elevation decreased by treating the rats with the FXR agonist. When compared with those rats in the sham group, unconjugated serum BAs in CLP rats were significantly increased. OCA administration significantly decreased the enhanced serum unconjugated BA concentration in CLP rats (Figure 4A). Both serum primary and secondary BAs increased after CLP administration. FXR activation by OCA reduced serum primary BA concentration in CLP rats (Figure 4B).

The proportion of unconjugated BAs in liver tissues was significantly higher in rats of the CLP group compared to those in the sham group and CLP + OCA group (Figure 4C). These alterations in serum and liver BAs showed support for FXR activation by OCA potentially normalizing the BA profile in CLP rats via repressing de novo BA synthesis in livers and promoting liver BA conjugation.

We then analyzed BA composition in liver tissues. As shown in Figure 4D, the abundance of CA, muricholic acid (α/β-MCA), CDCA, DCA, and 7-ketodeoxycholic acid (7-ketoDCA) increased in the livers of CLP rats, while GCA, GCDCA, and GUDCA decreased in rats of the CLP group compared with those of the sham group. OCA pre-treatment on CLP rats showed a moderating effect on each BA concentration to the level of sham-operated rats.

### 2.5. CA, DCA, and 7-ketoDCA Promote Inflammatory Phenotype in Rat Hepatocytes

**Figure 5:**
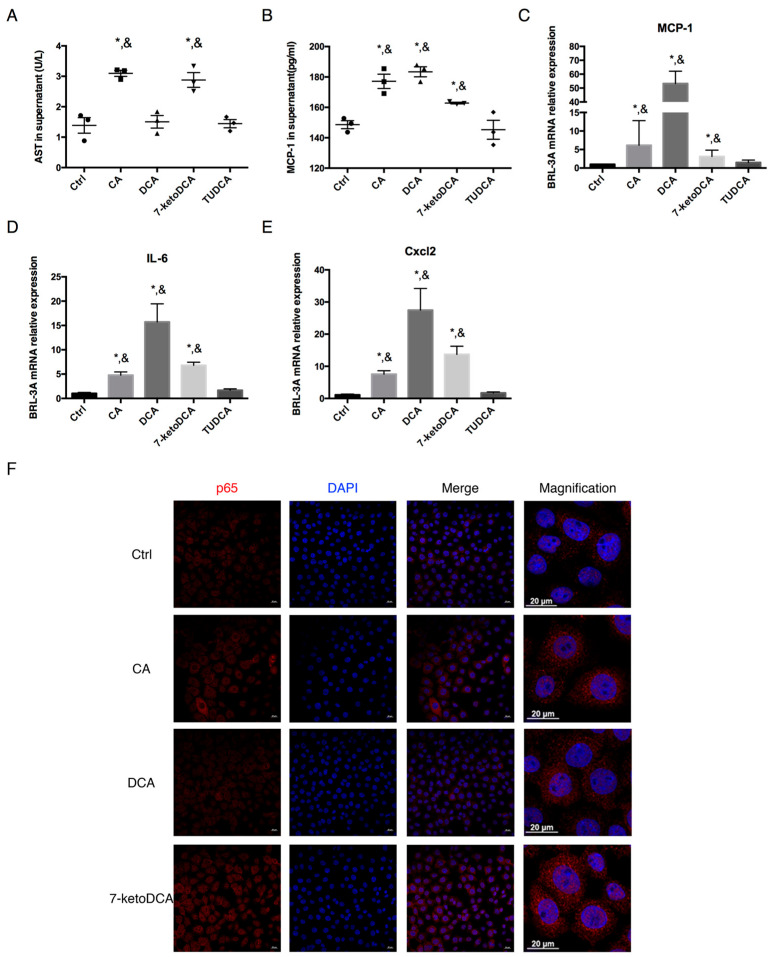
CA, DCA, and 7-ketoDCA promote inflammatory phenotype in BRL-3A cell line. (**A**) AST release and (**B**) MCP-1 secretion in BRL-3A supernatant co-cultured with 100 μM CA, DCA, 7-ketoDCA, and TUDCA separately for 6 h. BRL-3A mRNA levels of (**C**) MCP-1, (**D**) IL-6, and (**E**) Cxcl2 after 6 h of co-culturing. (**F**) Representative p65 (red) staining in BRL-3A cell slides counterstained with DAPI, scale bars = 20 μm. *, *p* < 0.05 compared with sham group; &, *p* < 0.05 compared with TUDCA group.

To further investigate whether the FXR agonist inhibits inflammation in the liver via regulating bile acids, the rat liver cell line BRL-3A was co-cultured with 100 μM CA, DCA, and 7-ketoDCA separately, as these BAs increased significantly in the liver tissues of CLP rats, while FXR activation by OCA decreased the elevation. In addition, 100 μM tauroursodeoxycholic acid (TUDCA) was used as a negative control. After 6 h of co-culture, 100 μM CA and 7-ketoDCA caused a 2-fold increase in the AST levels in BRL-3A cell supernatant (Figure 5A). CA, DCA, and 7-ketoDCA elevated the protein level of MCP-1 in the cell supernatant (Figure 5B). BRL-3A exposed to CA, DCA, and 7-ketoDCA showed significant increases in the mRNA expression of MCP-1, IL-6, and Cxcl2 (Figure 5C–E), whereas TUDCA in the same concentration barely induced any hepatic cell damage and pro-inflammatory mRNA expression. Immunofluorescence staining showed upregulation and nuclear translocation of the p65 subunit of NF-κB in BRL-3A cells after co-culturing with CA, DCA, and 7-ketoDCA.

## 3. Discussion

Sepsis-induced liver injury is related to high mortality [2,4,31]. Our study demonstrated that hepatic FXR activation attenuates both systemic and hepatic inflammation levels in septic rats induced by CLP operation, which are attributable to an improvement in bile acid metabolism and a reduction in elevated unconjugated BAs in the liver tissues of septic rats. The alteration of BA components such as CA, DCA, and 7-ketoDCA may aggravate liver inflammation and dysfunction via the activation of the NF-κB signaling pathway.

The present study focuses on FXR regulation and downstream bile acid metabolism alteration in sepsis. In the physiological condition, FXR directly upregulates the hepatocyte apical bile acid efflux transporter BSEP [32]. Also, FXR activates downstream SHP transcription and represses BA synthesis enzymes and NTCP [33]. These transcriptional activities ensure a decrease in BA retention and hepatocyte damage via promoting the canalicular export of BAs, and reducing BA synthesis and hepatocyte BA uptake from portal circulation [34]. When sepsis occurs, NF-κB activation and the release of acute inflammatory cytokines including TNF-α and IL-1β inhibit FXR activity, leading to bile acid metabolism imbalance [35,36]. Repressed hepatic FXR and abnormal BA transport manifesting through decreased BSEP were found in critical patients according to liver biopsies [37]. In the liver, restoration of FXR expression might be essential for reactivating FXR in the regulation of bile acid homeostasis [38].

Obeticholic acid (OCA) is an effective FXR agonist and is widely applied in chronic liver disease studies such as non-alcoholic fatty liver disease (NAFLD) and primary biliary cholangitis (PBC) [39]. OCA was found to protect mice against LPS-induced liver injury and inflammation [40] and inhibit hepatic inflammation in thioacetamide-induced cirrhotic rats, resulting in decreased HSC activation and fibrosis [41]. It is a semisynthetic derivative of CDCA and does not interfere with BA quantification using LC-MS/MS. Based on the pharmacokinetic studies in healthy subjects and NASH patients, the maximum plasma concentration (Cmax) time after a single 10 mg or 25 mg dose of OCA is 1.5 h [42,43]. Its quick peak time and safety characteristics make prophylactic administration realistic in clinical practice.

Our study confirms that a 10 mg/kg administration of OCA is able to activate hepatic FXR in CLP rats, thereby upregulating apical BSEP and NTCP. The latter allows a rapid transition of BAs from portal circulation to the bile duct, maintains a low hepatic intracellular BA concentration, and reduces circulating bile acids [44,45]. Different from previous studies showing that NTCP is repressed by FXR, our study presented a slight upregulation of NTCP in CLP rats with OCA pre-treatment, which was in accord with decreased serum BA concentrations. Apart from FXR, glucocorticoid receptor (GR), a nuclear steroid hormone receptor, as well as hepatocyte nuclear factor 1 (HNF1), possess NTCP transcriptional activation potential [32,46]. Here, we speculated that OCA upregulates NTCP in CLP rats through FXR-independent pathways, which requires further investigation.

During clinical practice, cholestasis rather than bile acid alteration is emphasized when evaluating sepsis-induced liver injury. However, an elevated bilirubin level reflects not only cholestasis but also hemolysis. Other than bilirubin, total bile acids may also serve as independent prognostic factors of liver dysfunction in sepsis [19]. In a clinical study in septic patients, circulating bile acids were elevated along with a deterioration of liver function [47]. Our study showed that OCA partially rescues the downregulation of FXR in experimental sepsis, reduces systemic inflammation and hepatic injury, and normalizes liver BA profiles. These results indicate that the modulation of BA metabolism may influence liver injury and outcome in septic patients. So far, no specific therapeutics for sepsis-induced liver dysfunction/failure are available. Whether the FXR agonist improves sepsis-induced liver dysfunction lacks relevant research. FXR activation with OCA could be a potential therapeutic target for cholestasis in sepsis.

Increasing evidence suggests a complex role of bile acids in inflammation apart from energy metabolism and gut barrier protection. The alteration of bile acid metabolism contributes to cholestatic liver diseases, inflammatory diseases in the digestive system, obesity, and diabetes [48]. Circulating bile acids are elevated in critically ill patients, induce immunosuppression, and are specific biomarkers predicting the mortality of septic patients [16,37,47]. In addition, hepatocytes release inflammatory chemoattractants, and cause neutrophil chemotaxis when BAs are at pathophysiological levels. A previous study indicated that bile acids act as ‘danger-associated molecular patterns’, activate the NLRP3 inflammasome in macrophages, and induce inflammation [38]. Excessive intracellular BAs result in mitochondrial injury and endoplasmic reticulum stress by activating Tlr9 and thereby initiate inflammatory response in the liver [49].

In the present study, UPLC/TQ-MS analysis enabled us to detect specific BA concentrations other than global profiling. Our study showed an obvious elevation of unconjugated BAs in both the serum and livers of CLP rats, while FXR activation decreased serum and hepatic unconjugated BA concentration in CLP rats. Notably, the total concentration of BAs did not differ significantly in CLP rat livers, indicating that not only the concentration but also the species of BA determine its pro-inflammatory and liver injury effects.

We observed the normalization of BA concentration in OCA-pre-treated CLP rats compared to CLP rats. It was revealed that the increased hepatic concentrations of CA, DCA, and 7-ketoDCA were downregulated by OCA in CLP rats. They are probably the main BA components causing hepatotoxicity in CLP rats as evidenced by a further in vitro mechanism study, in which these three BAs increased MCP-1 secretion and mRNA expression of MCP-1, IL-6, and Cxcl2 in the rat BRL-3A cell line, and was in line with a previous report on BA-induced hepatocyte injury [15]. The BRL-3A cell line has been widely used for toxic component testing [50] and MCP-1 is a typical biomarker significantly upregulated in mRNA and protein levels during the cellular inflammatory responses [51]. The pro-inflammatory effect of CA, DCA, and 7-ketoDCA was mediated by the NF-κB signaling pathway in BRL-3A cells. These results suggested that FXR activation may ameliorate liver injury partially via regulating bile acid homeostasis in CLP rats.

Clinical studies show increased serum-conjugated primary bile acids in septic shock patients compared to healthy controls [16,47]. Notably, the intestinal microbiota plays an important role in primary-to-secondary BA transformation and deconjugation [52]. Microbial diversity is impaired when sepsis occurs [53]. Secondary bile acids, specifically DCA and 7-ketoDCA, are steroidal bacterial metabolites produced in the colon derived from the conversion of the primary bile acid cholic acid (CA), carried by specific bile salt hydrolase (BSH)-expressing microorganisms [54]. Our study shows a significant elevation of 7-ketoDCA in the livers of CLP rats, which has rarely been studied previously. An in vitro study revealed that 7-ketoDCA rather than DCA is more likely to be formed in an acid medium (pH ≤ 7.0) by E. coli and some strains of bacteroides [55]. The decrease in the abundance of commensal *Parabacteroides distasonis* caused cecal 7-ketoDCA depletion, while the concentration of 12α-hydroxylated BAs (CA, DCA, and derivatives) increased in the serum and liver in non-sepsis calorie-restricted mice [56]. An imbalance in the BA pool and increased levels of these hydrophobic secondary bile acids may be attributable to the disrupted microbiome-mediated generation of 7-ketoDCA and the potential antibiotic confounders [57,58].

Our study innovatively specifies BAs with hepatotoxic manifestations, which exacerbate liver dysfunction via proinflammation pathways in sepsis. However, it still has some limitations. First of all, we did not test OCA after the CLP operation, which might be more relevant in guiding clinical practice. Secondly, a depletion of conjugated bile acids in CLP rat livers, including GCA, TCDCA, GCDCA, and GUDCA, was observed and requires further research, considering that CDCA and UDCA exhibit anti-inflammatory effects [59,60]. The regulative effect of the intestinal microbiota on bile acid metabolism and the involvement of FXR activation in sepsis also warrant further investigation.

## 4. Materials and Methods

### 4.1. Chemicals and Reagents

OCA, CA, and TUDCA were purchased from TargetMol Chemicals (TargetMol Chemicals Inc., Boston, MA, USA). 7-keto-deoxycholic acid was obtained from MCE (South Brunswick Township, NJ, USA). Deoxycholic acid was gained from Selleck (Houston, TX, USA). All BAs were dissolved in ethanol with final concentrations of the solvent less than 0.2%. Eagle’s Minimum Essential Medium (EMEM) and fetal bovine serum (FBS) were ordered from Lonsera (Ciudad de la Costa, Uruguay).

### 4.2. Animals and Treatments

The animal experiments protocol was approved by the local ethics committee under a project license granted by the Institutional of Animal Care and Use Committee of Shanghai Jiaotong University School of Medicine, and performed in accordance with the National Institutes of Health (NIH) guide for the care and use of laboratory animals.

In each experiment, a total of 36 Male Sprague Dawley (SD) rats (Zhejiang Vital River Laboratory Animal Technology Co., Ltd., Pinghu, China) weighing 240 to 250 g were kept on a regular 12 h light/dark cycle and received free access to water and food. Rats were randomly divided into four groups: (1) the sham group, N = 6; (2) the OCA group, N = 6; (3) the CLP group, with CLP 8 h, CLP 16 h, and CLP 24 h subgroups, N = 6 in each subgroup; and (4) the CLP + OCA group, N = 6. The rats were fasted overnight while maintaining free access to water before surgery. Rats in the OCA and CLP + OCA groups were administered with OCA (TargetMol Chemicals, Boston, MA, USA) by intragastric gavage (IG) of 10 mg/kg OCA in 5% ethanol as a vehicle. The dose of OCA chosen referred to a previous study [61]. Sepsis was induced in the CLP and CLP + OCA groups by CLP operation as detailed below. The operations were performed 2 h after OCA gavage. Rats in the sham group were sham-operated and treated with IG administration of the vehicle. The experiments and the following detection were biologically repeated three times.

### 4.3. Establishment of Sepsis Model by CLP Operation

Sepsis was induced by cecal ligation and puncture (CLP) as previously described [62,63]. Rats were anesthetized with 2% isoflurane. After disinfection and a 2.5 cm midline laparotomy in the abdomen, the cecum was exteriorized and ligated with a 3-0 silk ligature in the distal 1.5 cm. The ligated cecum was punctured twice with an 18 G needle and gently compressed until a small amount of stool appeared. The cecum was then returned and the abdomen was closed in two layers. A single laparotomy was performed in the sham-operated rats. All rats were resuscitated with saline (10 mL/kg) after operation subcutaneously. At 8 h, 16 h, and 24 h after CLP, these rats were euthanized separately. Their ileum and liver tissue specimens and serum samples were harvested and stored at −80 °C for further studies.

### 4.4. Cell Culture

The rat liver BRL-3A cell line (ATCC CRL-1442TM) was cultured in EMEM and supplemented with 10% FBS (Lonsera, Ciudad de la Costa, Uruguay) and 1% antibiotics (containing 100 units/mL penicillin and 100 μg/mL streptomycin) in a humidified atmosphere of 5% CO_2_ at 37 °C. Cells were treated with 100 μM CA, DCA, 7-ketoDCA, and TUDCA separately for 6 h. Cell supernatants, proteins, RNA samples, and cell slides were collected for corresponding analyses.

### 4.5. Histopathology, Immunohistochemical, and Immunofluorescence Staining

The ileum and liver tissues were fixed overnight in formalin and embedded in paraffin. Samples were sectioned into 3 μm thick slices for hematoxylin and eosin (H&E) staining and immunohistochemistry. The histopathological examination was conducted using an optical microscope and scored blinded by two pathologists independently. Intestinal mucosal damage was evaluated by Chiu’s scoring from 0 to 5 score according to intestinal mucosal pathological changes [64]. The liver injury was analyzed as previously described [65]. The ranking score ranged from 0 to 3 based on morphological changes.

Paraffin slices undergoing antigen retrieval were incubated with primary antibody against FXR (1:200, A9033a, Invitrogen, Carlsbad, CA, USA) overnight at 4 °C and thereafter with a HRP secondary antibody (Servicebio, Wuhan, China) at room temperature for 1 h. Afterwards, the slices were stained with DAB and hematoxylin counterstain (Servicebio, Wuhan, China) and analyzed with an optical microscope (Nikon Eclipse E100, NIKON, Shinagawa, Japan).

Collected BRL-3A cell slides were washed with PBS and permeabilized using Triton X-100 (Beyotime, Shanghai, China) for 15 min. After being blocked with 5% bovine serum albumin (Beyotime, Shanghai, China) for 30 min, the cell slides were stained with anti-p65 (polyclonal, 1:200, 10745-1-AP, Proteintech, Rosemont, IL, USA) overnight at 4 °C. Donkey anti-rabbit IgG Alexa Fluor^TM^ 555 (1:1000, A-31572, Invitrogen, USA) was used as a secondary antibody and cell nuclei were stained with DAPI (Servicebio, Wuhan, China). Immunostained samples were analyzed with a confocal microscope (Zeiss LSM 800, Zeiss, Oberkochen, German).

### 4.6. Western Blotting

Total protein of rat livers and BRL-3A cells was extracted with 1× cold RIPA lysis buffer containing protease and phosphatase inhibitors. The protein concentrations were quantificated and 20 μg per protein sample from BRL-3A cells and rat livers were subjected to 12.5% SDS-PAGE gel electrophoresis and transferred onto PVDF membranes (Millipore, Temecula, CA, USA). After blocking with blocking buffer (EpiZyme, Shanghai, China), the membranes were incubated in mouse monoclonal primary antibody against FXR (1:1000, A9033a, Invitrogen, USA) and rabbit monoclonal antibody against GAPDH (1:10,000, ab181602, Abcam, Cambridge, UK) overnight at 4 °C. After the membranes were incubated at room temperature for 1 h with polyclonal secondary antibodies (1:10,000, Jackson ImmunoResearch, West Grove, PA, USA), the blots were detected with ECL detection reagents and visualized using the GE ImageQuant LAS 4000 imaging system (Chicago, IL, USA).

### 4.7. Analysis of Biochemical Indicators

Rat serum was obtained by collecting arterial blood samples and centrifuging immediately at 3000 rpm for 15 min at 4 °C. Serum or cell supernatant alanine aminotransferase (ALT), aspartate aminotransferase (AST), total bile acid (TBA), and lactate dehydrogenase (LDH) were quantified using commercial assay kits (Rayto, Shenzhen, China) following the manufacturer’s instructions.

### 4.8. RNA Extraction and RT-qPCR

Total RNA from rat liver and BRL-3A cells was extracted using TRIzol reagent (Invitrogen, USA). After quality checking and quantification, 2 μg of total RNA per sample was converted to cDNA with PrimeScriptTM RT Master Mix (Takara, Mountain View, CA, USA) according to the manufacturer’s instructions. Real-time quantitative PCR (RT-qPCR) was performed in a 20 μL system containing 2 μL of cDNA sample and 0.3 μL each of 10μM forward and reverse primer) with a TB Green^®^ Premix Ex Taq™ II (Takara, Mountain View, CA, USA) using the StepOnePlus Real-time PCR system (Applied Biosystems, Thermofisher, Waltham, MA, USA). β-Actin was used as an internal control. Primers used for amplifications were obtained from Sangon Biotech Co., Ltd. (Shanghai, China). Information on the rat primer sequences is listed in Table 1.

### 4.9. Enzyme-Linked Immunosorbent Assay (ELISA)

Quantification of IL-1β, IL-6, and MCP1 in rat serum and the BRL-3A cell supernatant was measured with an ELISA kit (Mlbio, Shanghai, China) according to the manufacturer’s instructions. Absorbance was read at 450 nm using a TECAN Infinite^®^ 200 PRO (Männedorf, Switzerland) microplate reader. The investigator performing ELISA was unaware of group allocations.

### 4.10. Measurement of Bile Acids

Serum and liver BAs were assayed by liquid chromatography–tandem mass spectrometry (LC-MS/MS), using a SCIEX UPLC-Triple TOF 5600 system equipped with an ACQUITY HSS T3 column (100 mm × 2.1 mm i.d., 1.8 μm; Waters, Milford, MA, USA), and coupled to a quadrupole-time-of-flight mass spectrometer (Triple TOFTM5600+, Sciex, Framingham, MA, USA) equipped with an electrospray ionization (ESI) source.

### 4.11. Statistical Analysis

Statistical calculations were performed using Graph Pad Prism 6. Quantitative values with normal distribution are expressed as mean ± Standard Error of Mean (S.E.M.). Statistical analysis was performed using an unpaired Student’s *t*-test, one-way ANOVA, or Mann–Whitney U test, as appropriate. Bile acids were assessed by the Kruskal–Wallis H test and demonstrated with log. The statistical difference was *p* < 0.05.

## 5. Conclusions

Liver inflammation and dysfunction during sepsis is attributable to significant changes in bile acid composition in blood and liver. FXR activation reduces systemic inflammation and liver dysfunction by regulating bile acid homeostasis. Liver bile acid component alteration, more so than cholestasis, is an evident event in sepsis, which may aggravate liver inflammation and toxicity. FXR activation reduces systemic inflammation and liver injury partially by reducing cholestasis and toxic unconjugated BAs (CA, DCA, and 7-ketoDCA) in the liver. The protective role of FXR on BAs and the disrupted BA homeostasis in the development of gastro-hepatoxicity in sepsis provide novel insights on targeting FXR and BA-related pathways against sepsis-associated organ injuries, which warrant further basic and clinical studies.

## Figures and Tables

**Table 1:** Primers used in this work.

Gene	Forward Primer (5′-3′)	Reverse Primer (5′-3′)
β-actin	GCTGTGCTATGTTGCCCTAGACTTC	GGAACCGCTCATTGCCGATAGTG
FXR	AGGATAGAGAGGCAGTGGAGAAGC	AGCGTGGTGATGGTTGAATGTCC
SHP	GGCACTATCCTCTTCAACCCAGATG	GGGCTCCAGGACTTCACACAATG
Bsep	TTTGTTGGAAGCAGTGGGTGTGG	TGGAACGGAGGAACTGAATGTTGAC
Ntcp	CTTACTGGCTACCTCCTCCCTGATG	TGCAGCTTGGATTGAGTTGGAAGAG
IL-6	ACTTCCAGCCAGTTGCCTTCTTG	TGGTCTGTTGTGGGTGGTATCCTC
MCP-1	CTCACCTGCTGCTACTCATTCACTG	CTTCTTTGGGACACCTGCTGCTG
CXCL2	ATGCTGTACTGGTCCTGCTCCTC	GTCACCGTCAAGCTCTGGATGTTC

## Data Availability

All the citations and data included in this manuscript are available upon request by contact with the corresponding author.
